# Circadian Clock Gene Bmal1 Regulates Bilirubin Detoxification: A Potential Mechanism of Feedback Control of Hyperbilirubinemia

**DOI:** 10.7150/thno.35773

**Published:** 2019-07-09

**Authors:** Shuai Wang, Yanke Lin, Ziyue Zhou, Lu Gao, Zemin Yang, Feng Li, Baojian Wu

**Affiliations:** 1 Reserach Center for Biopharmaceutics and Pharmacokinetics, College of Pharmacy, Jinan University, 601 Huangpu Avenue West, Guangzhou 510632, China; 2 Integrated Chinese and Western Medicine Postdoctoral research station, Jinan University, 601 Huangpu Avenue West, Guangzhou, China; 3 Guangzhou Jinan Biomedicine Research and Development Center, Jinan University, 601 Huangpu Avenue West, Guangzhou, China; 4 International Cooperative Laboratory of Traditional Chinese Medicine Modernization and Innovative Drug Development of Chinese Ministry of Education (MOE), College of Pharmacy, Jinan University, Guangzhou, 510632, China

**Keywords:** circadian clock, Bmal1, Rev-erbα, bilirubin, hyperbilirubinemia

## Abstract

Controlling bilirubin to a low level is necessary in physiology because of its severe neurotoxicity. Therefore, it is of great interest to understand the regulatory mechanisms for bilirubin homeostasis. In this study, we uncover a critical role for circadian clock in regulation of bilirubin detoxification and homeostasis.

**Methods**: The mRNA and protein levels of Bmal1 (a core clock gene), metabolic enzymes and transporters were measured by qPCR and Western blotting, respectively. Luciferase reporter, mobility shift and chromatin immunoprecipitation were used to investigate transcriptional gene regulation. Experimental hyperbilirubinemia was induced by injection of bilirubin or phenylhydrazine. Unconjugated bilirubin (UCB) and conjugated bilirubin were assessed by ELISA.

**Results**: We first demonstrated diurnal variations in plasma UCB levels and in main bilirubin-detoxifying genes *Ugt1a1* and *Mrp2*. Of note, the circadian UCB levels were antiphase to the circadian expressions of Ugt1a1 and Mrp2. Bmal1 ablation abrogated the circadian rhythms of UCB and bilirubin-induced hepatotoxicity in mice. Bmal1 ablation also decreased mRNA and protein expressions of both Ugt1a1 and Mrp2 in mouse livers, and blunted their circadian rhythms. A combination of luciferase reporter, mobility shift, and chromatin immunoprecipitation assays revealed that Bmal1 trans-activated *Ugt1a1* and *Mrp2* through specific binding to the E-boxes in the promoter region. Further, Bmal1 ablation caused a loss of circadian time-dependency in bilirubin clearance and sensitized mice to chemical induced-hyperbilirubinemia. Moreover, bilirubin stimulated Bmal1 expression through antagonism of Rev-erbα, constituting a feedback mechanism in bilirubin detoxification.

**Conclusion**: These data supported a dual role for circadian clock in regulation of bilirubin detoxification, generating circadian variations in bilirubin level via direct transactivation of detoxifying genes Ugt1a1 and Mrp2, and defending the body against hyperbilirubinemia via Rev-erbα antagonism. Thereby, our study provided a potential mechanism for management of bilirubin related diseases.

## Introduction

Many aspects of physiology and behaviors in mammals are subjected to circadian rhythms 1. Circadian rhythms are driven by the circadian clock which is a transcriptional-translational feedback loop system 2. BMAL1 (brain and muscle ARNT-like 1) heterodimerizes with CLOCK (circadian locomotor output cycles kaput) [or NPAS2 (neuronal PAS domain protein 2)] and binds to the E-boxes to activate the transcription of clock-controlled genes (CCGs) including PER (period) and CRY (cryptochrome). Once reaching a threshold level, PER and CRY proteins form a heterodimer to inhibit the transcriptional activity of BMAL1-CLOCK/NPAS2, thereby repressing their own expressions and the expressions of other CCGs 3. This feedback loop system generates circadian oscillations in genes expressions and in many physiological and biochemical processes such as hormone secretion, cell differentiation and metabolism 4.

Bilirubin is a toxic end-product of heme catabolism in the body 5,6. High levels of free bilirubin (or unconjugated bilirubin, UCB) causes jaundice (hyperbilirubinemia), commonly seen in newborn children. The jaundice can lead to damages to the brain and even deaths 7. Bilirubin is detoxified mainly in the liver that involves multiple steps 8. UCB is transported into the hepatocytes by the organic anion transporters (OATP1B1/1B3 for humans and Oatp1a/1b for mice) 9,10. In hepatocytes, UCB is metabolized by UDP-glucuronosyltransferase (UGT) 1A1 to bilirubin mono- and di-glucuronides (i.e., conjugated bilirubin, CB). CB is then excreted into the bile by the efflux transporter multidrug-resistance protein 2 (MRP2/ABCC2) and into the blood circulation by MRP3 (ABCC3) for renal clearance 11. Hepatic UGT1A1 and MRP2 are critical determinants to bilirubin hemostasis. Genetic deficiency of UGT1A1 or MRP2 is associated with various forms of hyperbilirubinemia such as Crigler-Najjar, Gilbert and Dubin-Johnson syndromes 12,13.

Many xenobiotic-metabolizing enzymes (XMEs) [e.g., cytochrome P450 (CYP) 2a5, Cyp2b10 and sulfotransferase (SULT) 1a1] and drug transporters (e.g., P-glycoprotein) are oscillating genes [[14]-[15][16][17]]. Circadian variations in expressions of these genes underlie the chronopharmacokinetics, contributing to circadian time-dependent drug tolerance and/or toxicity 18. Circadian gene expressions of XEMs and transporters are generated directly by clock genes or indirectly by clock output genes 19. For instance, Bmal1 controls circadian Sult1a1 via direct transactivation 16. Circadian rhythms of Cyp2a5 and 2b10 are respectively accounted for by rhythmic nuclear receptors Ppar-γ and Car 15,20. Previous studies reported circadian variations in hepatic expressions of mouse Ugt1a1 and Mrp2 21,22. However, whether and how circadian clock controls UGT1A1 and MRP2 remain unclarified.

Many endogenous substances (e.g., melatonin, bile acids, glucocorticoid, glucose and fatty acids) in mammals are subjected to circadian rhythms. The rhythmic behaviors of these substances are necessary for normal physiological functions, and disruption of the rhythms is usually associated with various types of diseases 4,23,24,25. For instance, loss of a rhythm in melatonin release leads to disrupted energy metabolism and obesity 23. Disturbance of circadian bile acid metabolism can result in hepatic stress responses and liver injury 4. Although bilirubin is generally regarded as a neurotoxic compound, it may be biologically beneficial because of anti-oxidative and anti-inflammatory properties 26. An early clinical study reveals a diurnal variation in plasma bilirubin in humans. The circadian pattern of bilirubin is altered in individuals with abnormal sleep 27. This suggests that bilirubin is under the control of circadian clock. However, the molecular mechanism for regulation of bilirubin by circadian clock is unknown.

In this study, we investigated a potential role for circadian clock in regulation of bilirubin detoxification and homeostasis. We first demonstrated diurnal variations in mouse plasma bilirubin level and in main bilirubin-detoxifying genes *Ugt1a1* and *Mrp2*. The circadian plasma bilirubin levels were inversely correlated with circadian expression of Ugt1a1 and Mrp2. Further, Bmal1 controlled circadian Ugt1a1 and Mrp2 via direct transcriptional activation. Bmal1 ablation abrogated circadian time-dependent bilirubin clearance and sensitized mice to hyperbilirubinemia. Moreover, bilirubin up-regulated Bmal1 expression through antagonism of Rev-erbα, constituting a feedback mechanism in bilirubin detoxification. Our data established circadian clock as a critical regulator of bilirubin detoxification and homeostasis, thereby providing a novel mechanism for management of bilirubin related diseases.

## Results

### Bmal1 ablation blunts the circadian rhythms of unconjugated bilirubin (UCB) and bilirubin-induced toxicity

**Figure 1:**
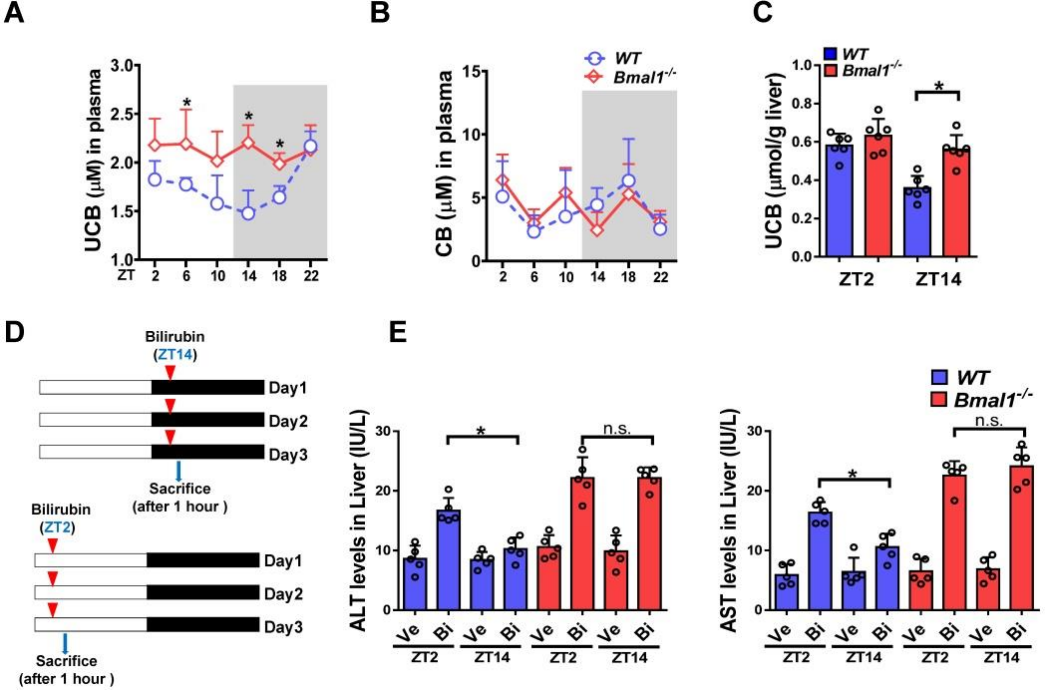
** Bmal1 ablation blunts the circadian rhythms of UCB and bilirubin-induced toxicity.** (A) Measurements of UCB in plasma from WT and *Bmal1^-/-^* mice. Data are mean ± SD (*n*= 6). *P < 0.05 for two group comparisons at individual time points (post hoc Bonferroni test). (B) Measurements of CB in plasma from WT and *Bmal1^-/-^* mice. Data are mean ± SD (*n*= 6). (C) Measurements of UCB in livers from WT and *Bmal1^-/-^* mice at ZT2 and ZT14. Data are mean ± SD (*n*= 6). *P < 0.05 (t test). (D) Schematic diagram for experimental protocol of bilirubin-induced hepatotoxicity. (E) Measurements of ALT and AST in plasma from* WT* and *Bmal1^-/-^* mice treated with bilirubin at ZT2 or ZT14. Data are mean ± SD (*n*= 5). *P < 0.05 (t test). Ve: vehicle; Bi: bilirubin. n.s.: not significant.

Plasma UCB level displayed a significant diurnal fluctuation in wild-type mice with a nadir value at ZT14 (Figure 1A). By contrast, the plasma level of conjugated bilirubin (CB) showed a weak fluctuation (Figure 1B). Bmal1 ablation increased plasma UCB in mice and abolished its circadian rhythm (Figure 1A). We also observed increased UCB level in the liver (Figure 1C). However, Bmal1 ablation had no effects on plasma CB (Figure 1B). Repeated injections of bilirubin (in three consecutive days, Figure 1D) to mice induced hepatotoxicity. The hepatotoxicity was dosing-time dependent with a more severe toxicity at ZT2 than at ZT14 (Figure 1E). Bmal1 ablation abrogated the dosing time-dependency of bilirubin-induced hepatotoxicity (Figure 1E). Collectively, these data indicated a critical role for Bmal1 in circadian regulation of bilirubin.

### Identification of cycling genes involved in bilirubin detoxification

**Figure 2:**
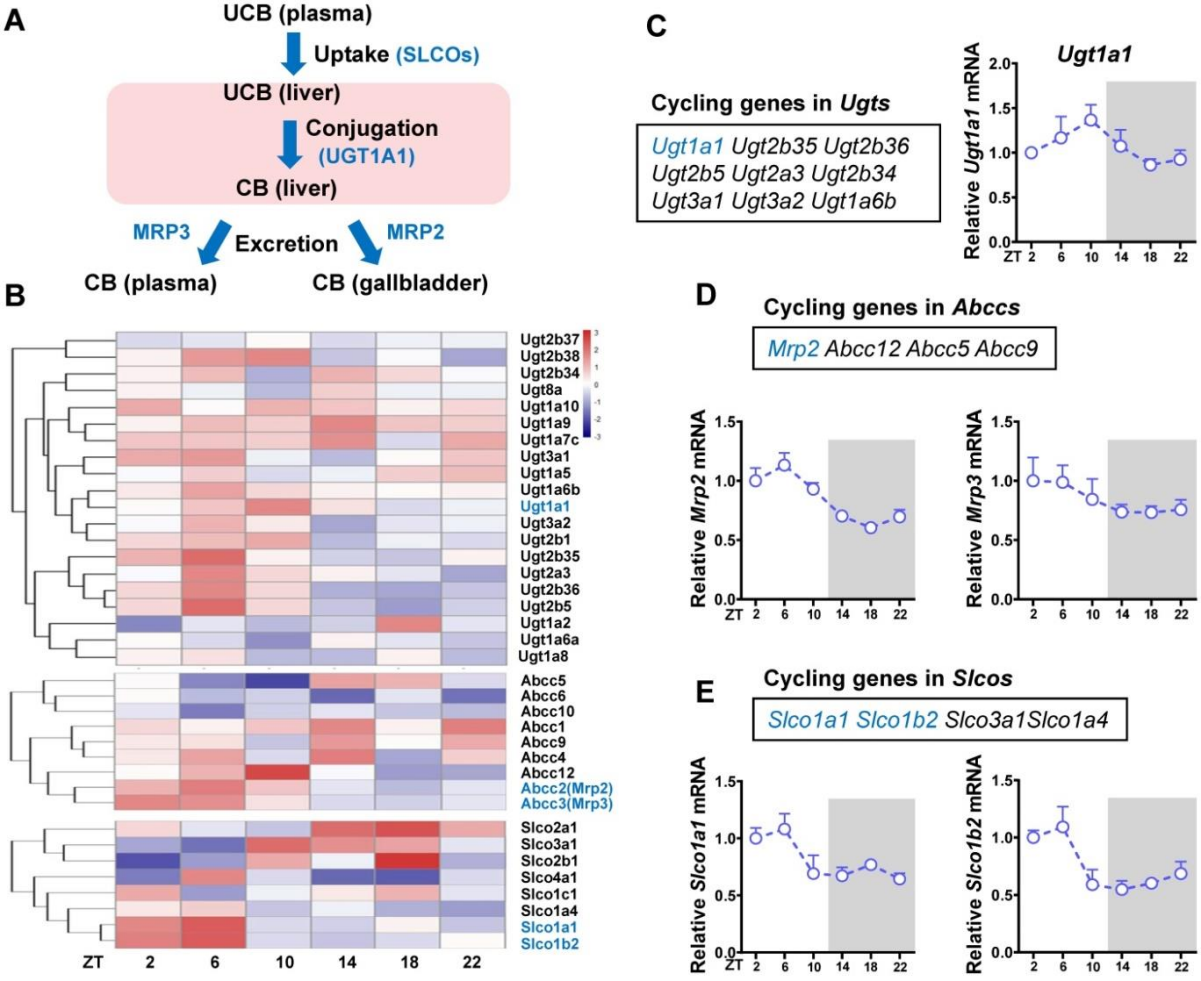
** Identification of cycling genes involved in bilirubin detoxification.** (A) The schematic diagram showing the detoxification processes of bilirubin *in vivo*. (B) Hierarchical clustering heatmap, showing gene expressions in livers of WT mice at different time points. Each row represents a gene and each column represents colon samples from different time points. Red indicates high relative expression and blue indicates low expression of genes as shown in the scale bar. (C) Cycling genes in Ugt enzymes family (left panel). Fragments per kilobase of transcript per million mapped (FPKM) of *Ugt1a1* at six time points (right panel). (D) Cycling genes in Abcc family (top panel). FPKM of Mrp2 and Mrp3 at six time points (bottom panel). (E) Cycling genes in Slco family (top panel). FPKM of Slco1a1 and Slco1b2 at six time points (bottom panel).

The liver is the major organ for bilirubin detoxification. The metabolic enzymes and transporters involved in liver detoxification of bilirubin include Ugt1a1, Mrp2 (Abcc2), Mrp3 (Abcc3), Slco1a (Oatp1a) and Slco1b (Oatp1b) (Figure 2A). RNA sequencing data revealed a number of genes from Ugt (*n* = 9), Abcc (*n* = 4) and Slco (*n* = 8) families as cycling genes (Figure 2B-E). Of note, *Ugt1a1* mRNA showed a robust diurnal fluctuation with a peak value at ZT10 (Figure 2C). *Mrp2, Slco1a1* and *Slco1b2* showed mild diurnal oscillations (Figure 2D-E). By contrast, *Mrp3* was a non-cycling gene (Figure 2D). The cycling genes *Ugt1a1*, *Mrp2, Slco1a1* and *Slco1b2* involved in bilirubin detoxification were selected for further studies.

### Bmal1 controls circadian expressions of Ugt1a1 and Mrp2

**Figure 3:**
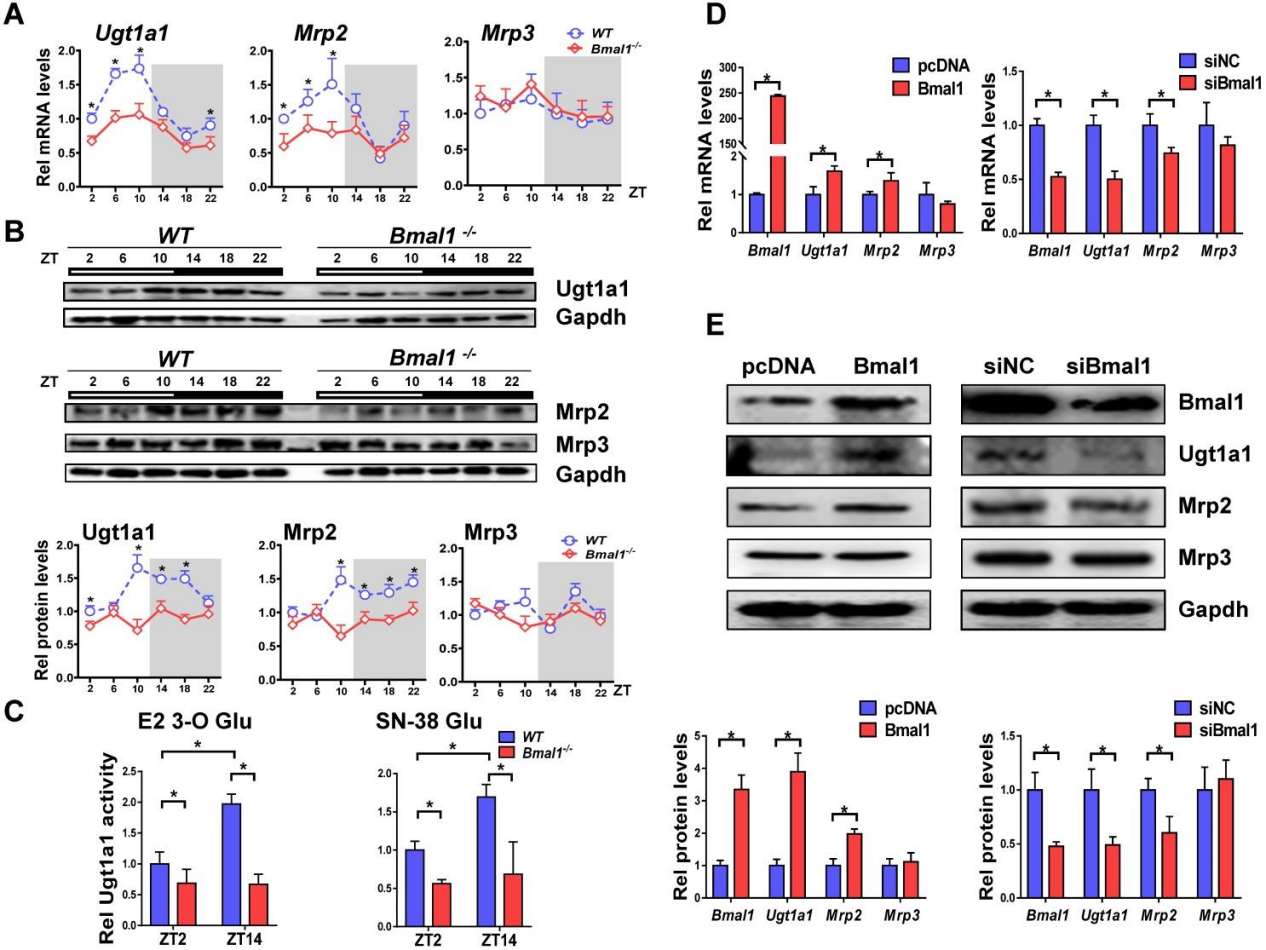
** Bmal1 regulates Ugt1a1 and Mrp2 expression.** (A) Rhythmic mRNA levels of* Ugt1a1, Mrp2* and* Mrp3* in the livers of WT and *Bmal1^-/-^* mice measured by qPCR. Data are mean ± SD (*n*=5). *P < 0.05 for two group comparisons at individual time points (post hoc Bonferroni test). (B) Rhythmic protein levels of Ugt1a1, Mrp2 and Mrp3 in the livers of WT and *Bmal1^-/-^* mice measured by Western blotting (top panel). The quantification data of Ugt1a1, Mrp2 and Mrp3 proteins bands (bottom panel). Data are mean ± SD (*n*=5). Statistical differences between blot density levels were analyzed by post hoc Bonferroni test. *P < 0.05 for two group comparisons at individual time points. (C) Liver microsomal metabolism assay showing decreased Ugt1a1 activity in livers of *Bmal1^-/-^* mice at ZT2 and ZT14. Data are mean ± SD (*n*=5). *P < 0.05 (post hoc Bonferroni test). (D) qPCR measurements of mRNA expressions for *Bmal1*, *Ugt1a1, Mrp2* and* Mrp3* in Hepa1-6 cells. Cells were transfected with Bmal1 overexpression plasmid or siRNA of Bmal1/negative control. (E) Bmal1, Ugt1a1, Mrp2 and Mrp3 protein expressions measured by Western blotting in Hepa1-6 cells transfected with Bmal1 overexpression plasmid or siRNA of Bmal1/negative control (top panel). The quantification data of Bmal1, Ugt1a1, Mrp2 and Mrp3 proteins bands (bottom panel). In panel D&E, data are mean ± SD (n=5). *P < 0.05 (t test).

Bmal1 ablation decreased mRNA and protein expressions of both Ugt1a1 and Mrp2 in mouse livers, and blunted their circadian rhythms (Figure 3A-B). By contrast, the expressions of *Abcc3*, *Slco1a1* and *Slco1b2* were unaffected (Figure 3A & Supplementary Figure 1). Liver microsomal metabolism assay revealed reduced glucuronidation of estradiol and SN-38 (two prototypical substrates of Ugt1a1) in *Bmal1^-/-^* mice consistent with the changes in Ugt1a1 protein (Figure 3C). Moreover, the circadian time difference in estradiol and SN-38 glucuronidation ceased to exist because of Bmal1 knockout (Figure 3C). Overexpression of Bmal1 in Hepa1-6 cells led to increases in mRNA and protein levels of both Ugt1a1 and Mrp2 (Figure 3D). Knockdown of Bmal1 caused reductions in Ugt1a1 and Mrp2 expressions (Figure 3E). By contrast, Mrp3 expression remained unchanged in these cell experiments (Figure 3D/E). Taken together, Bmal1 positively regulated Ugt1a1 and Mrp2 expressions, and was responsible for their circadian rhythms.

### Bmal1 trans-activates *Ugt1a1* and* Mrp2*

**Figure 4:**
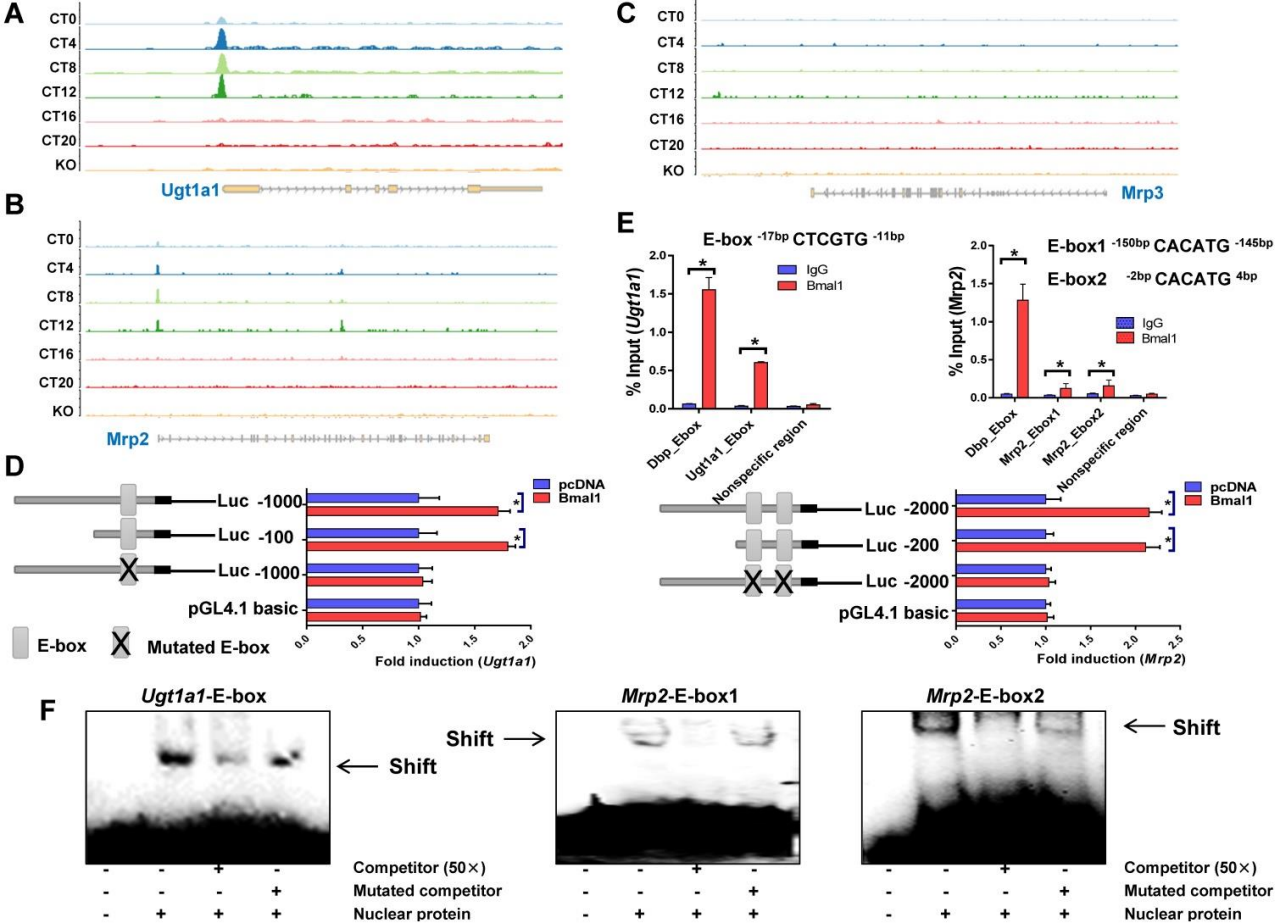
** Bmal1 regulates *Ugt1a1* and *Mrp2* transcription.** ChIP-sequencing for circadian Bmal1 binding to *Ugt1a1* (A), *Mrp2* (B) and *Mrp3* (C). Peaks indicate regions of DNA bound by Bmal1. ChIP-sequencing traces were generated from GSE39977. (D) Luciferase reporter assays with Hepa1-6 cells, showing the effects of Bmal1 on the activities of different versions of *Ugt1a1* promoters [i.e., *Ugt1a1* (-1000-bp~+100-bp), *Ugt1a1* (-100-bp~+100-bp) and *Ugt1a1* mutant (-1000-bp~+100-bp)], *Mrp2* promoters [i.e., *Mrp2* (-2000-bp~+100-bp), *Mrp2* (-200-bp~+100-bp) and *Mrp2* mutant (-2000-bp~+100-bp)] and pGL4.1 basic vector. Data are mean ± SD (*n*=6). *P < 0.05 (t test). (E) ChIP assays showing interactions of Bmal1 with *Ugt1a1* and *Mrp2* promoters in the livers of wild-type mice at ZT2. Immunoprecipitated chromatin was measured by qPCR with primers specific for the regions of *Dbp, Ugt1a1 and Mrp2*. Data are mean ± SD (*n*=5). *P < 0.05 (t test). (F) EMSA assays indicating that Bmal1 binds to E-boxes in the region of *Ugt1a1* promoter (-17-bp~-12-bp) and in the region of *Mrp2* promoter (-150-bp~-145-bp and -2-bp ~+4-bp).

ChIP sequencing analysis suggested circadian time-dependent binding of Bmal1 to the promoters of *Ugt1a1 and Mrp2,* and no binding to *Mrp3, Slco1a1 or Slco1b2* promoter (Figure 4A-C & Supplementary Figure 2). *In silico* algorithm (Genomatix) predicted one non-canonical E-box (-17- to -12-bp, a putative motif for Bmal1 binding) in *Ugt1a1* promoter. Bmal1 induced the luciferase reporter activity driven by the 100- or 1000-bp* Ugt1a1* promoter through the predicted E-box, supporting transcriptional regulation of *Ugt1a1* by Bmal1 (Figure 4D). ChIP assays showed significant recruitment of Bmal1 to the predicted E-box of *Ugt1a1* promoter in mouse liver (Figure 4E). EMSA assays confirmed a direct interaction of Bmal1 with the predicted *Ugt1a1* E-box (Figure 4F). *In silico* prediction suggested two non-canonical E-boxes (-150- to -145-bp and -2- to +4-bp) in the proximal region of *Mrp2* promoter. Direct binding of Bmal1 to these two E-boxes to activate Mrp2 transcription was validated by using luciferase reporter, ChIP and EMSA assays (Figure 4D-F). Collectively, these data indicated that Bmal1 trans-activated *Ugt1a1* and *Mrp2* through specific binding to the E-boxes in the promoter region.

### Bmal1 ablation sensitizes mice to hyperbilirubinemia

**Figure 5:**
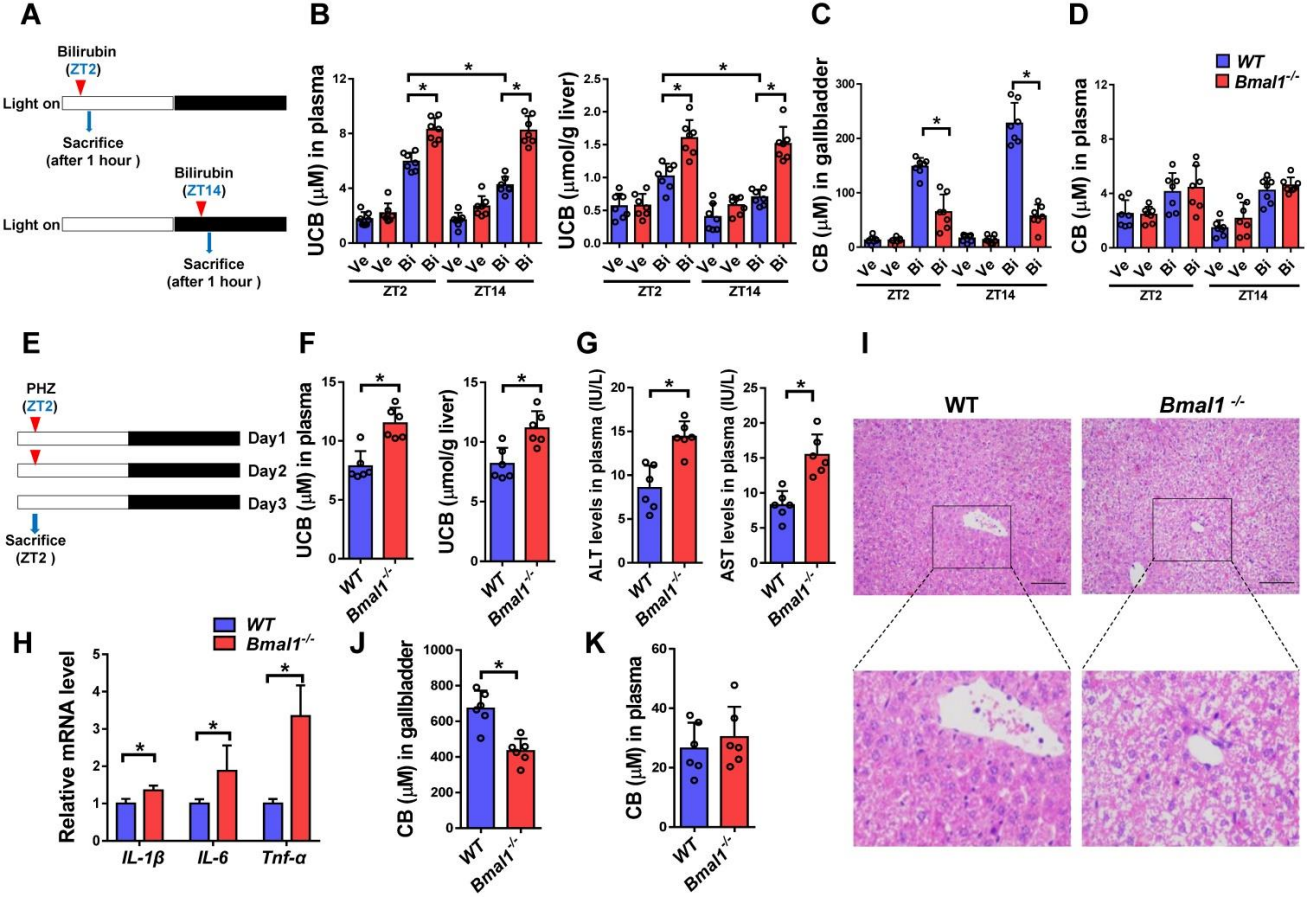
** Bmal1 ablation sensitizes mice to hyperbilirubinemia** (A) Schematic diagram for the experimental protocol of bilirubin-induced hyperbilirubinemia in WT and *Bmal1^-/-^*mice. (B) Measurements of UCB in plasma and livers of bilirubin or vehicle treated mice at ZT2 or ZT14. Data are mean ± SD (*n*=7). *P < 0.05 (post hoc Bonferroni test). (C) Measurements of CB in gallbladder of bilirubin or vehicle treated mice at ZT2 or ZT14. Data are mean ± SD (*n*=7). *P < 0.05 (t test). (D) Measurements of CB in plasma of bilirubin or vehicle treated mice at ZT2 or ZT14. Data are mean ± SD (*n*=7). (E) Schematic diagram for the experimental protocol of phenylhydrazine-induced hyperbilirubinemia in WT and *Bmal1^-/-^*mice. (F) Measurements of UCB in plasma and livers of phenylhydrazine-induced hyperbilirubinemia mice. (G) Measurements of ALT and AST in plasma of phenylhydrazine-induced hyperbilirubinemia mice. (H) Measurements of *IL-1β*, *IL-6* and *Tnfα* mRNA levels in livers of phenylhydrazine-induced hyperbilirubinemia mice. (I) Representative histopathological image of livers from hyperbilirubinemia mice induced by phenylhydrazine. (J) Measurements of CB in gallbladder of phenylhydrazine-induced hyperbilirubinemia mice. (K) Measurements of CB in plasma and livers of phenylhydrazine-induced hyperbilirubinemia mice. In panel F, G, H, J&K, data are mean ± SD (*n*=6). *P < 0.05 (t test). Ve: vehicle; Bi: bilirubin.

Intraperitoneal single injection of bilirubin at ZT2 or ZT14 induced hyperbilirubinemia in both wild-type and *Bmal1^-/-^* mice (Figure 5A/B). However, hyperbilirubinemia was more severe (significantly higher levels of plasma and liver UCB) in *Bmal1^-/-^* than in wild-type mice (Figure 5B). Exacerbated hyperbilirubinemia was associated with diminished production and biliary excretion of CB (Figure 5C). Undifferenced alterations in plasma CB level were observed between wild-type and *Bmal1^-/-^* mice after bilirubin treatment (Figure 5D). Moreover, bilirubin-induced hyperbilirubinemia was more severe at ZT2 than ZT14 in wild-type mice consistent with lower expressions of Ugt1a1 and Mrp2 (two bilirubin detoxification proteins) at ZT2 than ZT14 (Figure 5B & Figure 3B). Similar dosing time-dependent bilirubin clearance was observed in mice injected with bilirubin for three consecutive days (Supplementary Figure 3). However, the circadian time differences in hyperbilirubinemia ceased to exist in *Bmal1^-/-^* mice consistent with equal protein levels of Ugt1a1 (and Mrp2) at both circadian time points in the genetically modified mice (Figure 5B & Figure 3B).

We also assessed the role of Bmal1 in phenylhydrazine (PHZ)-induced hyperbilirubinemia (Figure 5E). Similarly, Bmal1 ablation sensitized mice to PHZ-induced hyperbilirubinemia (high levels of plasma and liver UCB in *Bmal1^-/-^* mice) (Figure 5F). The more severe hyperbilirubinemia agreed well with a higher level of hepatotoxicity (i.e., higher hepatic levels of ALT, AST and inflammatory cytokines) in *Bmal1^-/-^* mice (Figure 5G-H). This was further confirmed by histopathological analysis that showed higher incidences of hepatocellular swelling and degeneration in *Bmal1^-/-^* mice (Figure 5I). In addition, Bmal1 ablation caused a reduction in biliary excretion of CB, but had no effects on plasma CB (Figure 5 J & K).

### Feedback regulation of bilirubin by Bmal1

**Figure 6:**
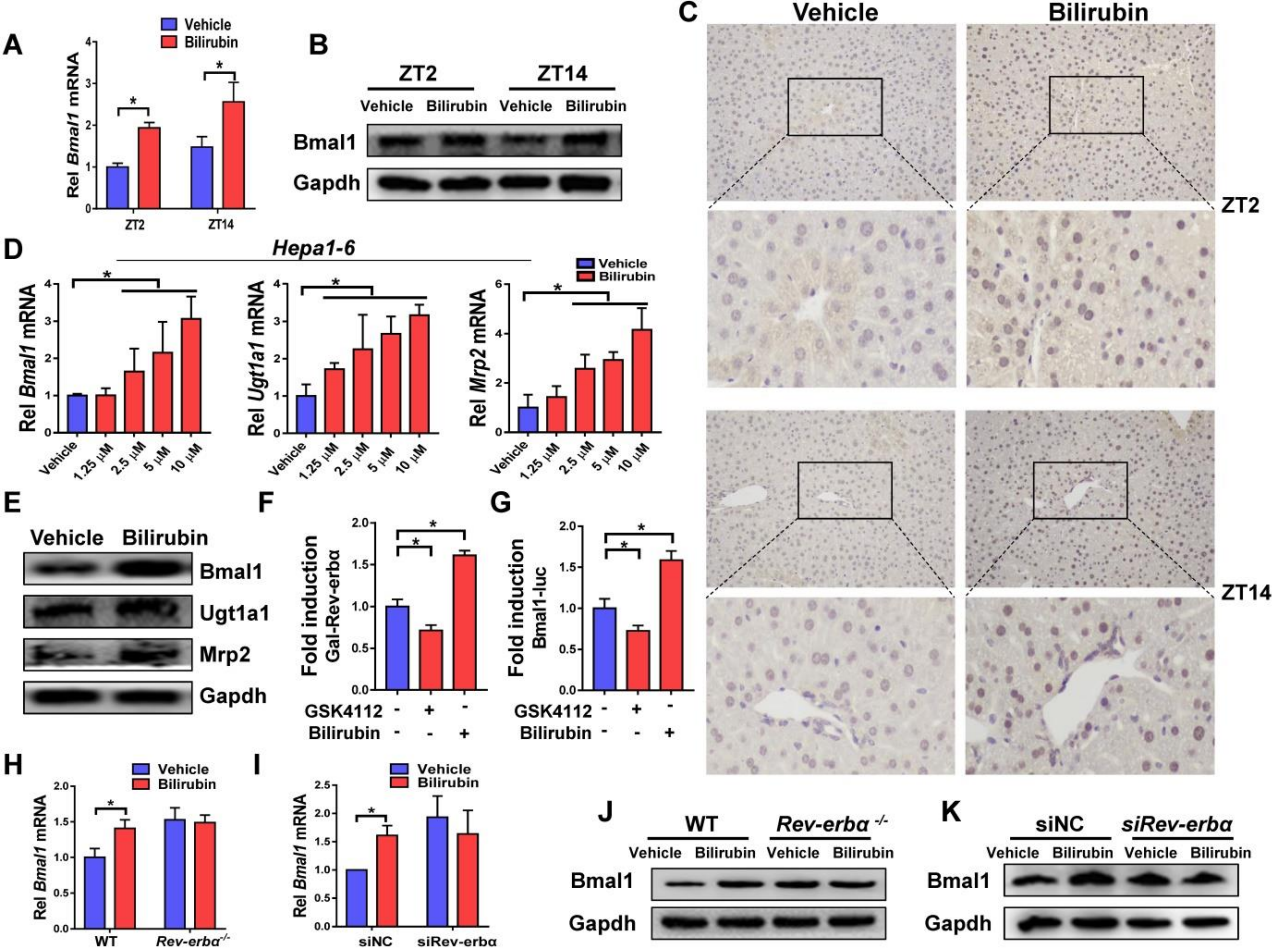
** A feedback regulation of bilirubin by Bmal1 through antagonism of Rev-erbα.** (A) Measurements of *Bmal1* mRNA in livers of WT mice by qPCR. Mice were treated with bilirubin or vehicle for 4 hours at ZT2 or ZT14. Data are mean ± SD (*n*=5). *P < 0.05 (t test). (B) Measurements of Bmal1 protein in livers of WT mice by Western blotting. Mice were treated with bilirubin or vehicle for 4 hours at ZT2 or ZT14. (Statistical differences between blot density levels were analyzed by t test, Supplementary Figure 7). (C) Immunohistochemistry for Bmal1 in livers of WT mice injected with bilirubin or vehicle at ZT2 and ZT14. (D) qPCR measurements of mRNA levels of *Bmal1*, *Ugt1a1* and *Mrp2* in Hepa1-6 cells. Data are mean ± SD (*n*=5). *P < 0.05 (post hoc Bonferroni test). (E) Protein expressions of Bmal1, Ugt1a1 and Mrp2 in Hepa1-6 cells measured by Western blotting. (Statistical differences between blot density levels were analyzed by t test, Supplementary Figure 7). (F) The effects of GSK4112 or bilirubin on the GAL4-Rev-erbα LBD reporter activity in Hepa1-6 cells. Data are mean ± SD (*n*=5). *P < 0.05 (post hoc Bonferroni test). (G) The effects of GSK4112 or bilirubin on mBmal1-promoter (-2000~+100-bp) reporter activity in Hepa1-6 cells. Data are mean ± SD (*n*=5). *P < 0.05 (post hoc Bonferroni test). (H) qPCR measurements of *Bmal1* mRNA in livers of WT and *Rev-erbα^-/-^* mice. Mice were treated with bilirubin or vehicle for 4 hours at ZT2. Data are mean ± SD (*n*=5). *P < 0.05 (t test). (I) qPCR measurements of *Bmal1* mRNA in Hepa1-6 cells transfected with siRNA of Rev-erbα or negative control. Data are mean ± SD (*n*=5). *P < 0.05 (t test). (J) Measurements of Bmal1 protein in livers of WT and *Rev-erbα^-/-^* mice by Western blotting. Mice were treated with bilirubin or vehicle for 4 hours at ZT2. (Statistical differences between blot density levels were analyzed by t test, Supplementary Figure 7). (K) Measurements of Bmal1 protein in in Hepa1-6 cells by Western blotting. Cells were transfected with siRNA of Rev-erbα or negative control. (Statistical differences between blot density levels were analyzed by t test, Supplementary Figure 7).

Injection of bilirubin to mice induced mRNA and protein expressions of Bmal1 in the liver (Figure 6A-B). Induction of hepatic Bmal1 expression in mice was confirmed by immunohistochemistry staining (Figure 6C). Consistently, bilirubin dose-dependently increased the mRNA levels of *Bmal1*, *Ugt1a1* and *Mrp2* in Hepa1-6 cells (Figure 6D). Protein levels of Bmal1, Ugt1a1 and Mrp2 were also increased in bilirubin-treated cells (Figure 6E). GAL4-Rev-erbα LBD cotransfection assay identified bilirubin as an antagonist of Rev-erbα, a transcriptional repressor of Bmal1 (Figure 6F). As expected, the Rev-erbα agonist GSK4112 inhibited the Bmal1-luc reporter activity (Figure 6G). By contrast, bilirubin increased the reporter activity, confirming its action as a Rev-erbα antagonist (Figure 6G). We also observed induced expressions of known Rev-erbα target genes such as *E4bp4*, *Pck1*, *G6pase* and *Cyp4a14* after bilirubin treatment (Supplementary Figure 4). The activation effects of bilirubin on hepatic Bmal1 expression were lost in *Rev-erbα^-/-^* mice (Figure 6H/J). Likewise, bilirubin failed to induce Bmal1 expression in *Rev-erbα* silenced Hepa1-6 cells (Figure 6I/K). Collectively, these data indicated a feedback regulation of bilirubin by Bmal1 through antagonism of Rev-erbα.

## Discussion

In this study, we observed a circadian variation in plasma bilirubin. This variation was associated with circadian time-dependent bilirubin detoxification and circadian expressions of detoxifying genes (Ugt1a1 and Mrp2) (Figures 1 & 3). The plasma bilirubin level was high at circadian time points when Ugt1a1 and Mrp2 expressions were low (i.e., weaker bilirubin detoxifying ability), and was low when Ugt1a1 and Mrp2 expressions were high (i.e., stronger bilirubin detoxifying ability) (Figures 1 & 3). We further showed that the circadian clock gene *Bmal1* controlled the rhythmic expressions of Ugt1a1 and Mrp2 via direct transactivation, thus regulated the sensitivity of mice to chemical induced-hyperbilirubinemia (Figure 4). Moreover, bilirubin itself served as an “enhancer” for Bmal1 regulation of bilirubin detoxification through antagonism of Rev-erbα (Figure 6). All these data supported a tight control of bilirubin detoxification and hemostasis by circadian clock.

Although the detoxification process is a key determinant to body level of bilirubin, formation of bilirubin from heme may also play a role. We thus determined the expression of heme oxygenase-1 (Hmox1, a rate-limiting enzyme responsible for metabolism of heme to bilirubin) and biliverdin reductase A (BVRA, catalyzing the last reaction in bilirubin formation) at six circadian time points, and evaluated the effects of Bmal1 on* Hmox1* and *Blvrα* 8*. Hmox1* and *Blvrα* expressions were circadian time-independent, and unaffected in *Bmal1^-/-^* mice (Supplementary Figure 5). Therefore, the circadian rhythm of bilirubin was mainly determined by the detoxification rather than formation process.

Our data indicated that of bilirubin-processing genes, Ugt1a1 and Mrp2 play dominant roles in bilirubin detoxification. This agrees well with the literature that biliary excretion is the main route for bilirubin detoxification 28. Although Mrp3 plays a limited role in bilirubin detoxification, it contributes to basolateral excretion of CB (to blood circulation). This was evidenced by the fact that plasma level of CB was unaltered in *Bmal1^-/-^* mice contrasting with reduced total formation of and reduced biliary excretion of CB due to down-regulated Ugt1a1 and Mrp2 (Figures 1 & 5).

Constitutive androstane receptor (CAR) is a xenobiotic-response nuclear receptor that is reported to regulate expressions of UGT1A1 and MRP 28. CAR is a known circadian gene that is under the control of PAR bZip transcriptional factors (Dbp, Hlf and Tef) and contributes to rhythmic expression of Cyp2b10 20. Dbp, Hlf and Tef are three target genes of Bmal1 29. Therefore, although a direct mechanism has been validated for Bmal1 regulation of Ugt1a1 and Mrp2 (Figure 4), an indirect mechanism involving CAR cannot be excluded. The possible indirect mechanism awaits further investigations.

The circadian clock gene *Bmal1* controls rhythmic expressions of Ugt1a1 and Mrp2, generating diurnal oscillations in plasma bilirubin. On the other hand, bilirubin at a relatively high concentration (≥2.5 μM, above its physiological level) promoted its own detoxification through antagonism of Rev-erbα and induction of Bmal1 expression. Moreover, Bmal1 ablation sensitized mice to hyperbilirubinemia. Therefore, the circadian clock has a dual role in regulating bilirubin detoxification. The first role is to generate circadian variations in bilirubin level and the second is to defend the body against hyperbilirubinemia. The latter role appears to be rather important because controlling bilirubin to a low level is necessary in physiology [bilirubin is toxic at a high level but is potentially beneficial (showing lipid-lowering, antioxidant and anti-inflammatory properties) at a low level] [[30]-[31][32]].

Heme is a known endogenous ligand (agonist) of Rev-erbα 33. We showed bilirubin, a catabolic product of heme, functions as an antagonist of Rev-erbα. Despite being structurally related, bilirubin and heme demonstrate different types of actions on REV-ERBα (i.e., antagonist *vs*. agonist). Similar observations were noted for other structurally related compounds [e.g., SR8278 *vs*. GSK4112; cobalt protoporphyrin IX *vs*. heme] 34. The distinct actions were probably due to a high sensitivity of REV-ERBα activity to the conformational changes in the ligand-bound receptor complex 35. This was supported by the fact that slight modifications of ligand-bound REV-ERBα by redox conditions and small gasses cause ligand switching and changes in functional effects 35.

We identified Bmal1 as a transcriptional activator of Ugt1a1 and Mrp2 (Figure 4). Bmal1 generates the circadian rhythms of two target genes via its own rhythmicity. This is supported by the fact that Ugt1a1 and Mrp2 mRNAs show similar circadian pattern to that of Bmal1 protein (Supplementary Figure 6). Therefore, we provided the underlying mechanisms for diurnal expressions of Ugt1a1 and Mrp2 that were not resolved previously 21,22. It is noteworthy that E-boxes were found in promoter regions of human UGT1A1 (-57 bp/-62 bp) and MRP2 (+4 bp/+9 bp). There is a possibility that human UGT1A1 and MRP2 are rhythmically expressed, and regulated by BMAL1. However, whether human BMAL1 regulates circadian expressions of UGT1A1 and MRP2 awaits further investigations. In addition, whether BMAL1/ REV-ERBα can be targeted for the management of bilirubin-related disease in humans was not addressed in current study.

In summary, circadian clock has a dual role in regulating bilirubin detoxification, generating circadian variations in bilirubin level via direct transactivation of detoxifying genes *Ugt1a1* and *Mrp2*, and defending the body against hyperbilirubinemia via Rev-erbα antagonism. Our study established a tight link between circadian clock and bilirubin detoxification, and provided a potential mechanism for management of bilirubin related diseases.

## Materials and Methods

### Materials

Hepa1-6 cells were purchased from American Type Culture Collection (Manassas, VA). The assay kits for Alanine transaminase (ALT) and Aminotransferase (AST) were purchased from Jiancheng Bioengineering Institute (Nanjing, Jiangsu, China). The ELISA kits for unconjugated bilirubin (UCB), conjugated bilirubin (CB), IL-1β, IL-6 and TNF-α were purchased from Meimian Biotechnology (Yancheng, Jiangsu, China). BCA assay kit, cytoplasmic/nuclear protein extraction kit, and EMSA kit were purchased from Beyotime (Shanghai, China). JetPrime transfection kit was purchased from Polyplus Transfection (Ill kirch, France). ChIP kit was purchased from Cell Signaling Technology (Beverly, MA). RNAiso Plus reagent and PrimeScript RT Master Mix were purchased from Takara (Shiga, Japan). SYBR Green Master Mix was purchased from Vazyme (Nanjing, Jiangsu, China). Dual-Luciferase® Reporter Assay System was purchased from Promega (Madison, WI). Bilirubin was purchased from Aladdin Chemicals (Shanghai, China). The antibodies used for Western blotting were as follows: anti-Ugt1a1 (Abcam, Cambridge, MA), anti-Mrp2 (Proteintech, Chicago, IL), anti-Bmal1 (Abcam, Cambridge, MA), anti-Mrp3 (OriGene Technologies, Rockville, MD), and anti-Gapdh (Abcam, Cambridge, MA). The horseradish peroxidase-conjugated secondary antibody was purchased from Huaan Biotechnology (Hangzhou, Zhengjiang, China). For chromatin immunoprecipitation assay, anti-Bmal1 antibody was purchased from Abcam (Cambridge, MA). For immunohistochemistry assay, anti-Bmal1 antibody was purchased from Proteintech (Chicago, IL). Ugt1a1 and Mrp2 luciferase reporters and siRNAs were obtained from TranSheep (Shanghai, China). Bmal1 plasmid, Bmal1 luciferase reporter, GAL4-Rev-erbα LBD, GAL4-responsive luciferase reporter and pRL-TK were obtained from Biowit Technologies (Shenzhen,China).

### Animals studies

Wild-type (WT) C57BL/6 mice were obtained from Beijing HFK Bioscience (Beijing, China). *Bmal1^-/-^* mice and *Rev-erbα^-/-^*mice were generated on a C57BL/6 background as described in our previous publication 37. All mice were bred and maintained on a 12h L/12h D cycle (light on 7:00 AM to 7:00 PM) at 22-25°C, with access to food and water at the Institute of Laboratory Animal Science (Jinan University, Guangzhou, China). Protocols for animal experiments were approved by the Institutional Animal Care and Use committee of Jinan University (Guangzhou, China). In the first set of experiments (for assessment of bilirubin-induced hepatotoxicity), wild-type and *Bmal1^-/-^* mice (male) were injected with bilirubin (i.p., 60 mg/kg) once daily (at ZT2 or ZT14) for three consecutive days. Mice were sacrificed 1 hour after the last dosing. The plasma samples were collected and subjected to ALT and AST analyses. In the second set of experiments (for measurement of bilirubin clearance), bilirubin was injected via the tail vein at ZT2 or ZT14 to wild-type, *Bmal1^-/-^* and* Rev-erbα^-/-^* mice (male) as described previously 35. After 1 hour, the mice were sacrificed, followed by collection of the plasma, liver and gallbladder samples. To evaluate the effects of Bmal1 on hyperbilirubinemia development, wild-type and *Bmal1^-/-^*mice (male) were treated with phenylhydrazine (i.p., 75 mg/kg) once daily at ZT2 for two consecutive days. On day 3, mice were sacrificed at ZT2 for collection of plasma, liver and gallbladder samples. Liver tissues were fixed in 4% paraformaldehyde and embedded in paraffin, followed by hematoxylin-eosin (H&E) staining and imaging (Nikon digital sight DS-FI2, Tokyo, Japan).

### Cell experiments

Hepa1-6 cells were cultured in Dulbecco's Modified Eagle Medium (DMEM) supplemented with 10% fetal bovine plasma. After reaching a confluence of 60-70%, the cells were transfected with Bmal1 overexpression plasmid or siRNA using the JetPrime transfection kit according to the manufacturer's protocol. 24 h later, the cells were collected for qPCR or Western blotting. In another set of experiments, the cells were treated with bilirubin or vehicle. After 4 h, the cells were collected for qPCR or Western blotting.

### Quantitative polymerase chain reaction (qPCR) and RNA-sequecing

Total RNA was extracted from cell or liver samples using RNAiso Plus reagent. The reverse transcription was performed to obtain cDNA using the PrimeScript RT Master Mix. The cDNA was amplified on an ABI 7300 real time PCR system using the SYBR Green Master Mix as previously described 16. Relative expression was derived using the 2^-ΔΔCT^ method. Ppib was used as an internal control. The sequences of all primers are listed in Supplementary Table 1.

For RNA-sequencing, livers were collected at six time points (ZT2, ZT6, ZT10, ZT14, ZT18 and ZT22, *n*=3 per group) from mice under a 12h L/12h D condition. RNA was extracted, and the purity and concentration were measured by using Nanodrop 2000 (Thermo Fisher Scientific, Wilmington, DE). The integrity of RNA was assessed by using an Agilent 2100 bioanalyzer (Agilent Technologies, Santa Clara, CA). RNA with RIN (RNA Integrity Number) > 7.5 was used for construction of NEB libraries. The library preparations were sequenced on an Illumina Hiseq X TEN platform (Novogene, Beijing, China). Transcriptomic data were analyzed as previously described 37.

### Luciferase reporter assays

In the first set of assays, Hepa1-6 cells were transfected with the luciferase reporters (i.e., Ugt1a1-luc or Mrp2-luc), pRL-TK plasmid (an internal control with renilla luciferase gene), and Bmal1 overexpression plasmid or blank pcDNA using JetPrime transfection kit. 24 h after transfection, the cells were collected for luciferase activity measurements using the Dual-Luciferase^®^ Reporter Assay System and GloMax^TM^ 20/20 luminometer (Promega, Madison, WI). The firefly luciferase activity was normalized to renilla luciferase activity, and expressed as relative luciferase unit. In the second set of assays, 2.0 kb *Bmal1* reporter and pRL-TK were transfected into Hepa1-6 cells. 24 h later, the medium was changed to phenol-free DMEM containing GSK4112 (10 μM) and/or bilirubin (10 μM). After another 12 h, the cells were collected for the measurements of luciferase activities. GAL4-Rev-erbα LBD cotransfection assay was performed as previously described 36. In brief, Hepa1-6 cells were transfected with GAL4-Rev-erbα LBD, GAL4-responsive luciferase reporter TK-UAS-Luc and pRL-TK. 24 h later, the medium was changed to phenol-free DMEM containing GSK4112 (10 μM) and/or bilirubin (10 μM). After another 12 h, the cells were collected for the measurements of luciferase activities.

### Chromatin immunoprecipitation (ChIP)

ChIP assays were performed as previously described 37. In brief, mouse liver samples were cross-linked in 1% formaldehyde, followed by digestion (with micrococcal nuclease) and sonication. The sheared chromatin samples were incubated with anti-Bmal1 antibody or normal rabbit IgG (a negative control) overnight at 4℃. DNA was isolated from immunoprecipitates and subjected to qPCR with specific primers (Supplementary Table 2).

### Electrophoretic mobility shift assay (EMSA)

EMSA assays were performed using a chemiluminescent EMSA kit as described previously 37. In brief, nuclear proteins were extracted from Bmal1 transfected Hepa1-6 cells using cytoplasmic/nuclear protein extraction kit. The nuclear proteins were incubated with unlabeled probes (or unlabeled mutated probes), followed by adding the biotin-labeled probes (*Ugt1a1*-E-box, *Mrp2*-E-box1 or *Mrp2*-E-box2). The products were separated on a 5% polyacrylamide gel and transferred into the Hybond N+ nylon membranes. The protein-DNA complexes were visualized using enhanced chemiluminesence reagent and Omega Lum G imaging system (Aplegen, San Francisco, CA). The sequences of probes are listed in Supplementary Table 3.

### Western blotting

The cell and tissue samples were lysed in RIPA buffer containing 1% phenylmethanesulfonyl fluoride. The samples were subjected to 10% sodium dodecyl sulfate polyacrylamide gel electrophoresis, and transferred onto polyvinylidene difluoride membranes. The membranes were incubated with primary antibodies overnight, followed by incubation with horseradish peroxidase-conjugated secondary antibody. The protein bands were visualized using Omega Lum G imaging system (Aplegen), and quantified with densitometry using Quantity One software (Bio-Rad, Hercules, CA).

### Immunohistochemistry (IHC)

Mouse livers were fixed in 4% paraformaldehyde. Paraffin-embedded sections were dewaxed using xylene and rehydrated in ethanol. After boiling, samples were blocked with 5% goat serum and incubated using a monoclonal rabbit antibody against Bmal1 overnight, followed by incubation with the secondary goat anti-rabbit horseradish peroxidase antibody. One hour later, samples were stained with diaminobenzidine tetrahydrochloride and counterstained with hematoxylin. The images of these samples were obtained using a Nikon Eclipse Ti-SR microscope (Nikon Incorporation, Tokyo, Japan).

### Statistical analyses

Data are presented as mean ± SD, and were analyzed using GraphPad Prism 7 (GraphPad Software Inc., San Diego, CA). Statistical analyses for multiple groups were performed using one-way or two-way analysis of variance (ANOVA), followed by a post-hoc Bonferroni test. Statistical analyses for two groups were performed using Student's t-test. The level of significance was set at p < 0.05 (*). JTK_CYCLE algorithm was used to detect cycling genes with adjusted p values of smaller than 0.05.

## Supplementary Material

Supplementary figures and tables.

## Abbreviations

Bmal1/BMAL1: mouse/human brain and muscle ARNT-like 1
CAR: constitutive androstane receptor
CB: conjugated bilirubin
ChIP: chromatin immunoprecipitation assays
CRY: cryptochrome
DMEM: Dulbecco's Modified Eagle Medium/High glucose
DMSO: dimethyl sulfoxide
EMSA: electrophoretic mobility shift assay
FBS: fetal bovine plasma
Mrp2/MRP2: mouse/human multidrug-resistance protein 2
Mrp3/MRP3: mouse/human multidrug-resistance protein 3
PER: period
PHZ: phenylhydrazine
Ppar: Peroxisome proliferator-activated receptor
qPCR: real-time polymerase chain reaction
UCB: unconjugated bilirubin
Ugt1a1/ UGT1A1: mouse/human UDP-glucuronosyltransferase 1A1
WT: wild-type
