# Supplementary material for: Circadian Clock Gene Bmal1 Regulates Bilirubin Detoxification: A Potential Mechanism of Feedback Control of Hyperbilirubinemia
Source: Theranostics. 2019 Jul 9;9(18):5122–33. doi: 10.7150/thno.35773 (PMC6691581; doi:10.7150/thno.35773)
Supplement: Supplementary file 1 — Supplementary figures and tables. [file thnov09p5122s1.pdf]

Supplemental Information

Circadian clock gene Bmal1 regulates bilirubin detoxification: a potential mechanism of feedback control of hyperbilirubinemia

Supplementary Table 1. Mouse primer sequences for quantitative real-time PCR (qPCR)

Gene	Forward (5'-3' sequence)	Reverse (5'-3' sequence)
Bmal1	CTCCAGGAGGCAAGAAGATTC	ATAGTCCAGTGGAAGGAATG
Ugt1a1	CACTGGCTGAGTATGCTTGG	CTTCTGGAATGGCACAGGGAA
Mrp2	AGCCGACCTGCCTCTTTGTT	CAAGAGCCAAAGAAAGCCCAA
Mrp3	CTGCCCCAGACGGACTTTAT	TCTGGTGCGTAGTTTCGGAG
Sloc1a1	CAGGGGCATGCAGGATGTAT	GTCTGGAGAGTGGATGTCGC
Sloc1b2	CACCGACCAAAGCTGATTGG	AGAAATGTGGCAACGCAGTC
Homx1	CCTTCTGGTATGGGCCTCAC	CCTCGTGGAGACGCTTTACA
Blvra	ACGAGAACTTGGGAGCCTTG	AGGCGACATCAACCTCTTGG
E4bp4	CTTTCAGGACTACCAGACATCCAA	GATGCAACTTCCGGCTACCA
Pck1	ATGAAAGGCCGCACCATGTA	GCACAGATATGCCCATCCGA
G6pase	TGGTAGCCCTGTCTTTCTTTG	TTCCAGCATTCACACTTTCCT
Cyp4a14	CCCAAAGGTATCACAGCCACAA	CAGCAATTCAAAGCGGAGCAG
Ppib	CTCTCGGAGCGCAATATGAAG	GCAAAAGGAAGACGACGGAG

Supplementary Table 2. Primer sequences for ChIP assays.

Gene	Forward (5'-3' sequence)	Reverse (5'-3' sequence)
Ugt1a1-E-box	TCATGGGGCACAATCAATCA	ACGGAAGAAGCAGAAGCAAAC
Ugt1a1-nonspecific region	GCTGCCATCTTGAAACCCAG	AATCTCGGGTCCTTATGCTTGC
Mrp2-E-box-1	ACATCTCTGTGAACTCTTAACCG	TAATGCAGTTGGCAGGTCGG
Mrp2-E-box-2	GAGAAGTGATAAGCCTCCCAGT	GAATGCTATGTCATCCCAGTGAGC
Mrp2-nonspecific region	GTTCGCTATCACATTTCAG	CTCATTGCCTTCACTTCA

Supplementary Table 3. Oligonucleotide sequences for EMSA assays

Oligonucleotide	Forward (5'-3' sequence)	Reverse (5'-3' sequence)
Mrp2-E-box1(biotin)	TAAAACCCCACATGCAGGGAT	ATCCCTGCATGTGGGGTTTTA
Mrp2-E-box1(cold)	TAAAACCCCACATGCAGGGAT	ATCCCTGCATGTGGGGTTTTA
Mrp2-E-box1(mut)	TAAAACCCTGTGCACAGGGAT	ATCCCTGTGCACAGGGTTTTA
Mrp2-E-box2(biotin)	TCCCAGTCACATGTCTGCTCA	TGAGCAGACATGTGACTGGGA
Mrp2-E-box2(cold)	TCCCAGTCACATGTCTGCTCA	TGAGCAGACATGTGACTGGGA
Mrp2-E-box2(mut)	TCCCAGTAGTGCTTCTGCTCA	TGAGCAGAAGCACTACTGGGA
Ugt1a1-E-box(biotin)	TTCGGATGTTCCAGCTGCCTTTATCTCC	GGAGATAAAGGCAGCTGGAACATCCGAA
Ugt1a1-E-box(cold)	TTCGGATGTTCCAGCTGCCTTTATCTCC	GGAGATAAAGGCAGCTGGAACATCCGAA
Ugt1a1-E-box(mut)	TTCGGATGTTCATCGATCCTTTATCTCC	GGAGATAAAGGATCGATGAACATCCGAA

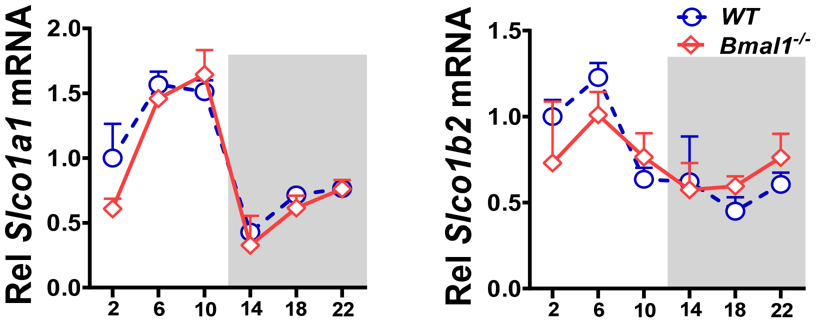

Supplementary Figure 1. Bmal1 ablation does not affect *Slco1a1* and *Slco1b2* expressions. (A) Circadian mRNA levels of *Slco1a1* and *Slco1b2* in the livers of WT and *Bmal1*$^{-/-}$ mice measured by qPCR. Data are mean ± SD (n=5).

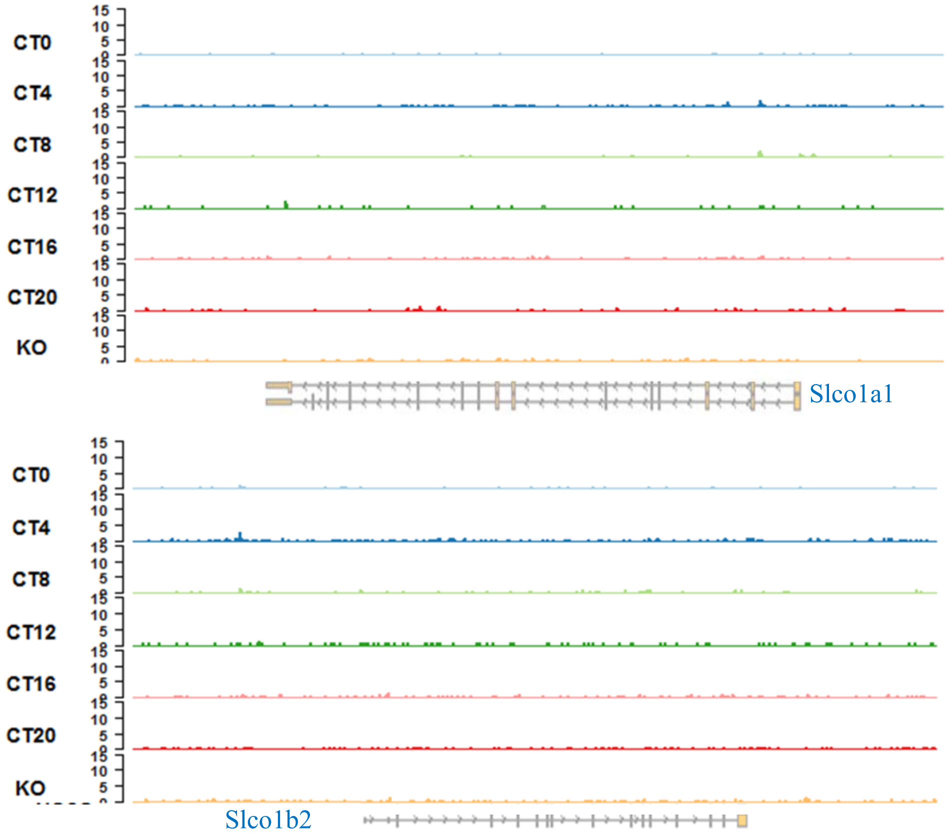

Supplementary Figure 2. ChIP-sequencing for circadian Bmal1 binding to *Slco1a1* and *Slco1b2.* No obvious peaks were found in the data. ChIP-sequencing traces were generated from GSE39977.

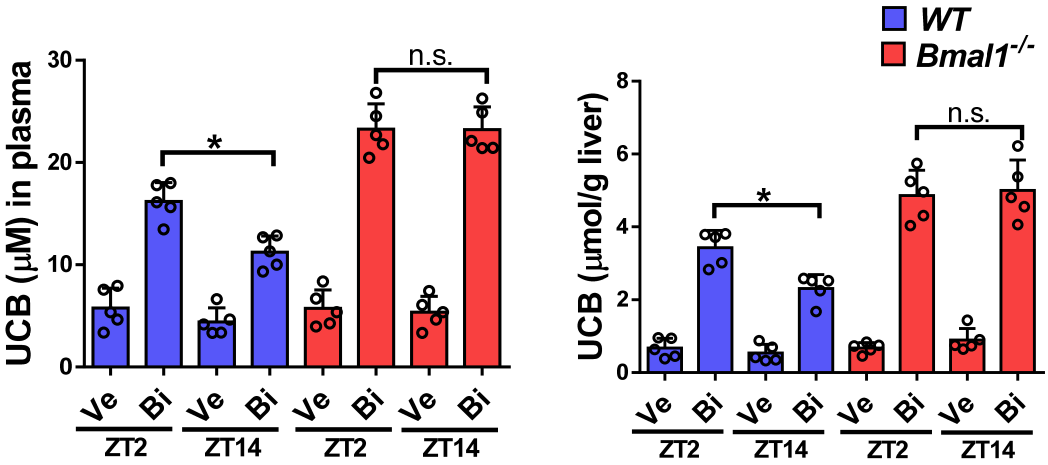

Supplementary Figure 3. Measurements of UCB in plasma and livers of bilirubin or vehicle-treated mice at ZT2 or ZT14. Data are mean ± SD (n=5). *P < 0.05 (t test). Ve: vehicle; Bi: bilirubin.

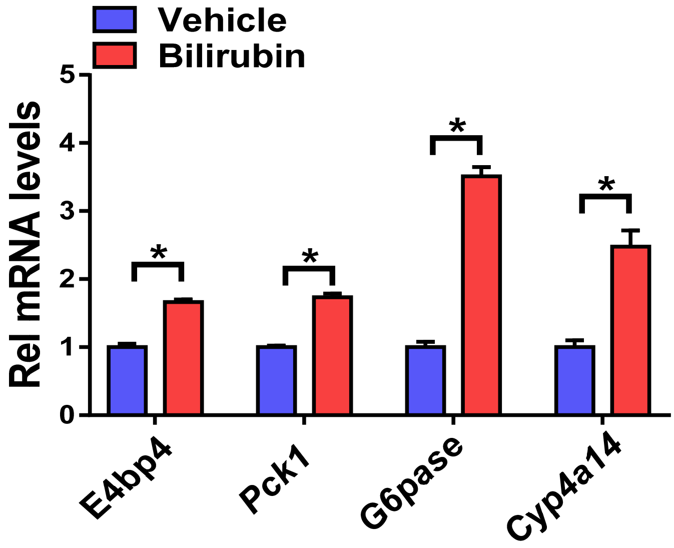

Supplementary Figure 4. qPCR measurements of mRNA levels of *E4bp4*, *Pck1*, *G6pase* and *Cyp4a14* in Hepa1-6 cells. Data are mean ± SD (n=5). *P < 0.05 (t test).

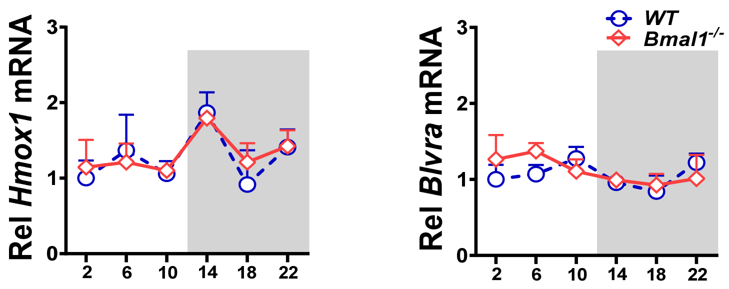

Supplementary Figure 5. Bmal1 ablation does not affect *Hmox1* and *Blvrα* expressions. (A) Circadian mRNA levels of *Hmox1* and *Blvrα* in the livers of WT and *Bmal1*$^{-/-}$ mice measured by qPCR. Data are mean ± SD (*n*=5).

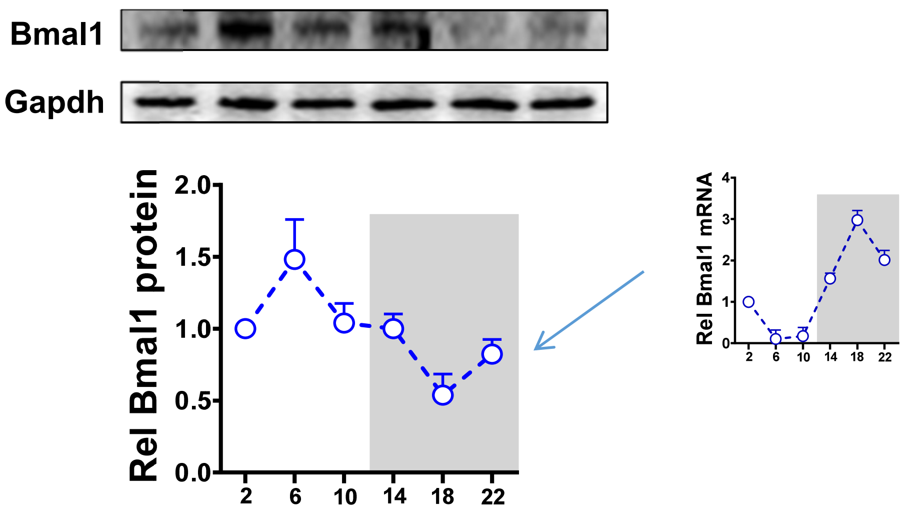

Supplementary Figure 6. Circadian protein (left panel) and mRNA (right panel) expression of Bmal1 in the livers of Wild-type mice.

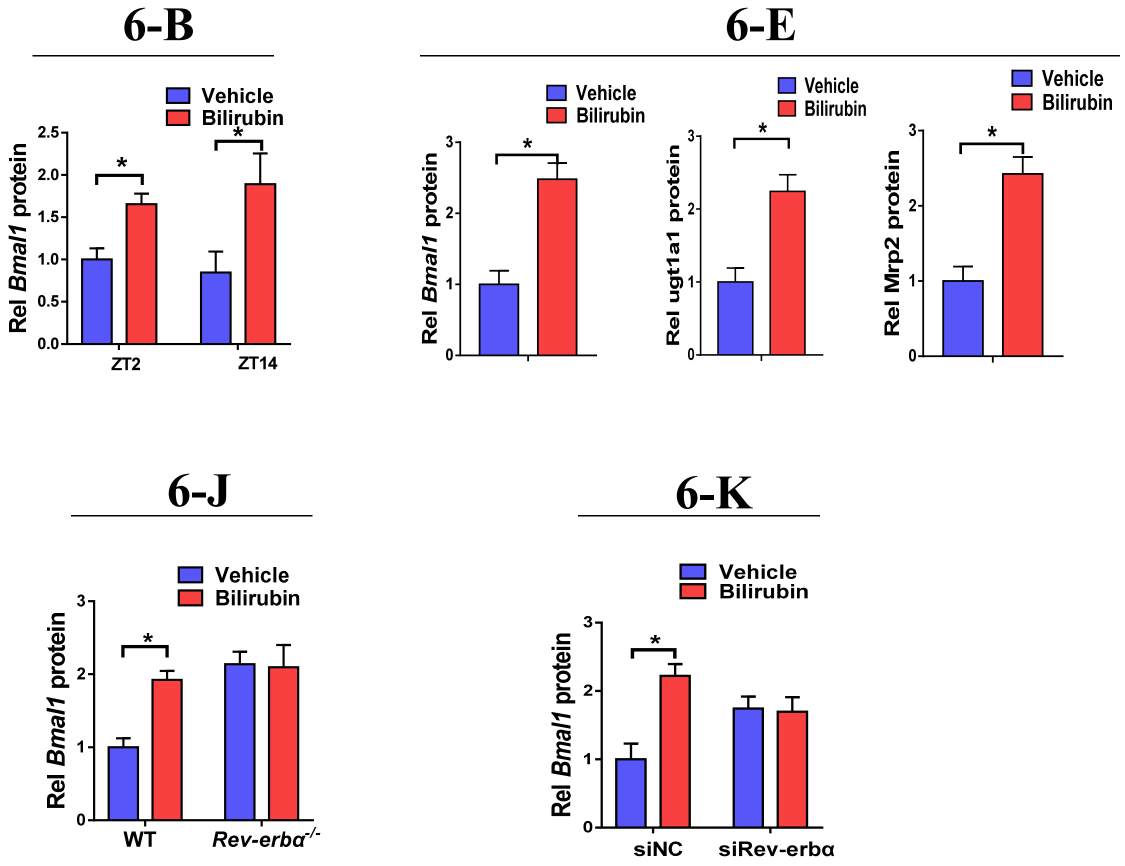

Supplementary Figure 7. Quantification of the blot band density for Figure 6.
